# Degeneration rather than hypoplasia may be the pathologic feature of the endolymphatic sac in patients with ipsilateral delayed endolymphatic hydrops

**DOI:** 10.3389/fneur.2026.1896403

**Published:** 2026-07-21

**Authors:** Kaijun Xia, Ping Lei, Qin Liu, Yingzhao Liu, Cen Chen, Xiaoye Chen, Xingqian Shen, Ziying Xu, Bo Liu, Hongjun Xiao, Yangming Leng

**Affiliations:** 1 Department of Otorhinolaryngology-Head and Neck Surgery, ENT Institute, Union Hospital, Tongji Medical College, Huazhong University of Science and Technology, Wuhan, China; 2 Department of Radiology, Union Hospital, Tongji Medical College, Huazhong University of Science and Technology, Wuhan, China; 3 Hubei Provincial Clinical Research Center for Precision Radiology & Interventional Medicine, Wuhan, China; 4 Hubei Province Clinical Center for Deafness and Vertigo, Wuhan, China

**Keywords:** angular trajectory of the vestibular aqueduct, delayed endolymphatic hydrops, endolymphatic Hydrops, endolymphatic sac, Ménière’s disease

## Abstract

**Objective:**

Delayed endolymphatic hydrops (DEH) and Ménière’s disease (MD) are both hydropic ear disease. In MD patients, the endotypes of MD are classified based on pathological types of the endolymphatic sac (ES), which can be distinguished by a radiological indicator: the angular trajectory of the vestibular aqueduct (ATVA). Studies have exhibited that MD patients of different pathological types of ES manifest different clinical characteristics. Here, we aimed to investigate the prevalence of each ATVA category in DEH and MD, and to further explore the clinical significance of ATVA in ipsilateral DEH patients.

**Methods:**

Twenty-seven ipsilateral DEH patients, 114 unilateral MD patients and 45 control participants were enrolled in the present study. The ATVA was determined using temporal bone CT imaging. Clinical features, including gender, age and the caloric test results, were collected. The prevalence of ATVA categories were compared between patients with ipsilateral DEH and unilateral MD. The association between ATVA and clinical features was also examined.

**Results:**

(1) Compared to MD-affected ears, ATVA ≤ 120° was more frequent (χ^2^ = 9.231, *p* = 0.002), whereas ATVA ≥ 140° was less frequent in DEH-affected ears (χ^2^ = 6.865, *p* = 0.009). (2) Compared to control ears, the distribution of ATVA categories in affected ears of ipsilateral DEH patients was not significantly different (χ^2^ = 1.190, *p* = 0.510). (3) The prevalence of ATVA ≥ 140°, 120° < ATVA < 140° and ATVA ≤ 120° in affected ears of ipsilateral DEH patients was comparable to that in non-affected ears (χ^2^ = 1.083, *p* = 0.568).

**Conclusion:**

Compared to unilateral MD patients, degenerative ES may be the dominant pathological type in ipsilateral DEH patients due to the high occurrence of ATVA ≤ 120°.

## 1 Introduction

Ménière’s disease (MD) is a chronic disorder of the inner ear characterized by episodic vertigo, fluctuating hearing loss, tinnitus, and/or ear fullness (1, 2). The pathological hallmark of MD is endolymphatic hydrops (ELH). MD patients exhibit high inter-individual variability (3), and MD subtyping holds significant clinical implications to etiological research and prognostic analysis. Frejo et al. (4) performed a two-step cluster analysis in MD patients, and defined five clinical variants of unilateral MD: classical MD, delayed MD, familial MD, sporadic MD plus migraine and autoimmune MD. The heterogenous clinical features support the clinical classification of MD (5–7).

Most recently, using immunohistochemistry and *in situ* protein–protein interaction detection assays in murine and human inner ears, Eckhard et al. (8) found that two pathological endotypes of endolymphatic sac (ES) are consistently present in patients with MD: hypoplastic and degenerative ES. By correlating radiographs and temporal bone specimens from patients with MD, Bächinger et al. (9) further demonstrated that the angular trajectory of the vestibular aqueduct (ATVA) was a radiographic surrogate indicator for these two ES pathologies. Typically, fetal vestibular aqueducts (VA) were nearly straight (ATVA ≥ 140°), whereas mature VA were curved (ATVA ≤ 120°). Juliano et al. (10) found ATVA transitions from a fetal (≥140°) to mature (≤120°) trajectory across the first decade. Then, it was suggested that, for the patients with MD, hypoplastic ES associated with abnormally straight VA (ATVA ≥ 140°) may be due to developmental factors, whereas degenerative ES associated with maturely curved VA (ATVA ≤ 120°) may be caused by postnatal factors (9). The relevance of symptom-based (phenotype) and pathology-based (endotype) classification has recently been investigated by Bächinger *et al*., who found that the prevalence of family history of inner ear disease (MD, hearing loss and/or vertigo) was higher in hypoplasia subgroup (41.2%) than in degeneration subgroup (15.7%), along with a male predominance and a tendency toward earlier disease onset (11), suggesting a significant genetic contribution in MD patients with hypoplastic ES. In addition, studies of ATVA in MD patients suggest that pathological endotypes of ES correlate to bilateral progression (12), severe to profound hearing loss (13), EH grade of cochlear and vestibular compartment (14), suggesting that ATVA has potential applications in the study of the pathogenesis and prognosis of MD.

Delayed endolymphatic hydrops (DEH) is a rare clinical entity with distinct manifestations from MD, although both disorders share a common histopathological feature of ELH (15, 16). DEH can be divided into two subgroups: ipsilateral and contralateral DEH (17). Ipsilateral DEH patients experience a precedent unilateral profound hearing loss and recurrent episodic vertigo of delayed onset. Contralateral DEH patients typically exhibit fluctuating hearing loss in the opposite better-hearing ear after a prolonged period of profound deafness, which may be associated with episodic vertigo attacks. Patients with a previous history of sensorineural hearing loss (months or years) before the onset of vertigo episodes are diagnosed as having delayed MD (4). Though Frejo et al. (4) consider delayed MD as a subtype of unilateral MD, the current available diagnostic criteria of DEH are different from those of delayed MD, which highlight a precedent profound sensorineural hearing loss, i.e., a pure tone average (PTA) of greater than 90 dB over the 0.5-kHz, 1.0-kHz, and 2.0-kHz frequencies (17). The symptoms of delayed MD are similar to those of DEH, but the hearing level is not explicitly defined in the criteria of delayed MD. Moreover, there is no clinical definition of ipsilateral and contralateral types of delayed MD. Recently, Chen et al. (18) studied the gene expression profile of DEH and MD by high-throughput RNA-Seq analysis, and revealed that DEH and MD are different manifestations of the same disease. In addition, some anatomical differences exist between these two subtypes of hydropic ear disease. The vertical part of the posterior semicircular canal - the posterior fossa distance (PPD) is usually accepted as a radiological measure of ES size (19). Lei et al. (20) found that PPD of the affected side in unilateral MD patients was shorter than that of ipsilateral DEH patients. Until now, the role of ATVA in MD pathogenesis and subtyping has been explored in several studies (9, 11–14, 21–25), whereas the clinical significance of ATVA in ipsilateral DEH patients remains elusive.

The aim of the study is to examine the distribution of the ATVA categories in patients with ipsilateral DEH and unilateral definite MD, and further explore the potential associations between ATVAs and clinical features in ipsilateral DEH patients.

## 2 Participants and methods

### 2.1 Participants

This retrospective study enrolled 27 ipsilateral DEH patients, 114 unilateral definite MD patients who attended the Department of Otorhinolaryngology-Head and Neck Surgery, Union Hospital, Tongji Medical College, Huazhong University of Science and Technology from 2016 to 2023. The diagnosis of ipsilateral DEH was established against the criteria formulated by the committee of the Japan Society for Equilibrium Research in 1987 (17). MD was diagnosed following the diagnostic guideline proposed by Classification Committee of the Bárány Society in 2015 (1). The exclusion criteria were: (1) middle or inner ear malformations; (2) middle or inner ear infections (otitis media, mastoiditis, labyrinthitis *etc*.); (3) contralateral DEH in the DEH group or bilateral MD in the MD group; (4) retro-cochlear lesions (vestibular schwannoma, internal acoustic canal stenosis *etc*.); (5) history of ear surgery or intratympanic injections; (6) systemic diseases; (7) other vestibular or balance disorders (vestibular migraine, multiple sclerosis, cerebellar infarction, *etc*.). Clinical stage of MD was defined according to pure-tone average (PTA) using the AAO-HNS criteria: stage 1, ≤25 dB; stage 2, 26–40 dB; stage 3, 41–70 dB; stage 4, >70 dB(2). The control group included 45 trauma patients who attended Union Hospital, Tongji Medical College, Huazhong University of Science and Technology during the same recruitment period (excluding ears with temporal bone fracture, previous otitis media, vertigo, or hearing loss).

This study was conducted in compliance with the tenets of the Declaration of Helsinki. Informed consent was obtained from every participant. The project was approved by the ethical committee of Tongji Medical College of Huazhong University of Science and Technology (Institutional Review Board approval [2023] No. 0354).

### 2.2 Radiological evaluation

#### 2.2.1 CT protocols

CT scans were performed utilizing a 64-detector spiral CT scanner (Somatom Definition AS+, Siemens, Forcheim, Germany). Participants in this research were positioned supine and scanned along the craniocaudal axis. The scanning plane was aligned with the orbitomeatal line. The following scan parameters were used: tube voltage 120 kV, CASE Dose 4D quality reference mAs: 180 mAs, slice thickness 0.6 mm, slice collimation 128 × 0.6 mm, pitch 0.5, field of view 150 mm, reconstruction increment 0.3 mm and reconstruction kernel H60s.

The CT images were subsequently transferred and processed on a picture archiving and communication system (PACS) workstation (Carestream Client, Carestream Health). Multiplane reconstructions were carried out in the axial, coronal, and sagittal orientations. The radiological assessments were conducted by two experienced neuroradiologists who were unaware of the patients’ clinical information.

#### 2.2.2 Radiological measurement

**Figure 1:**
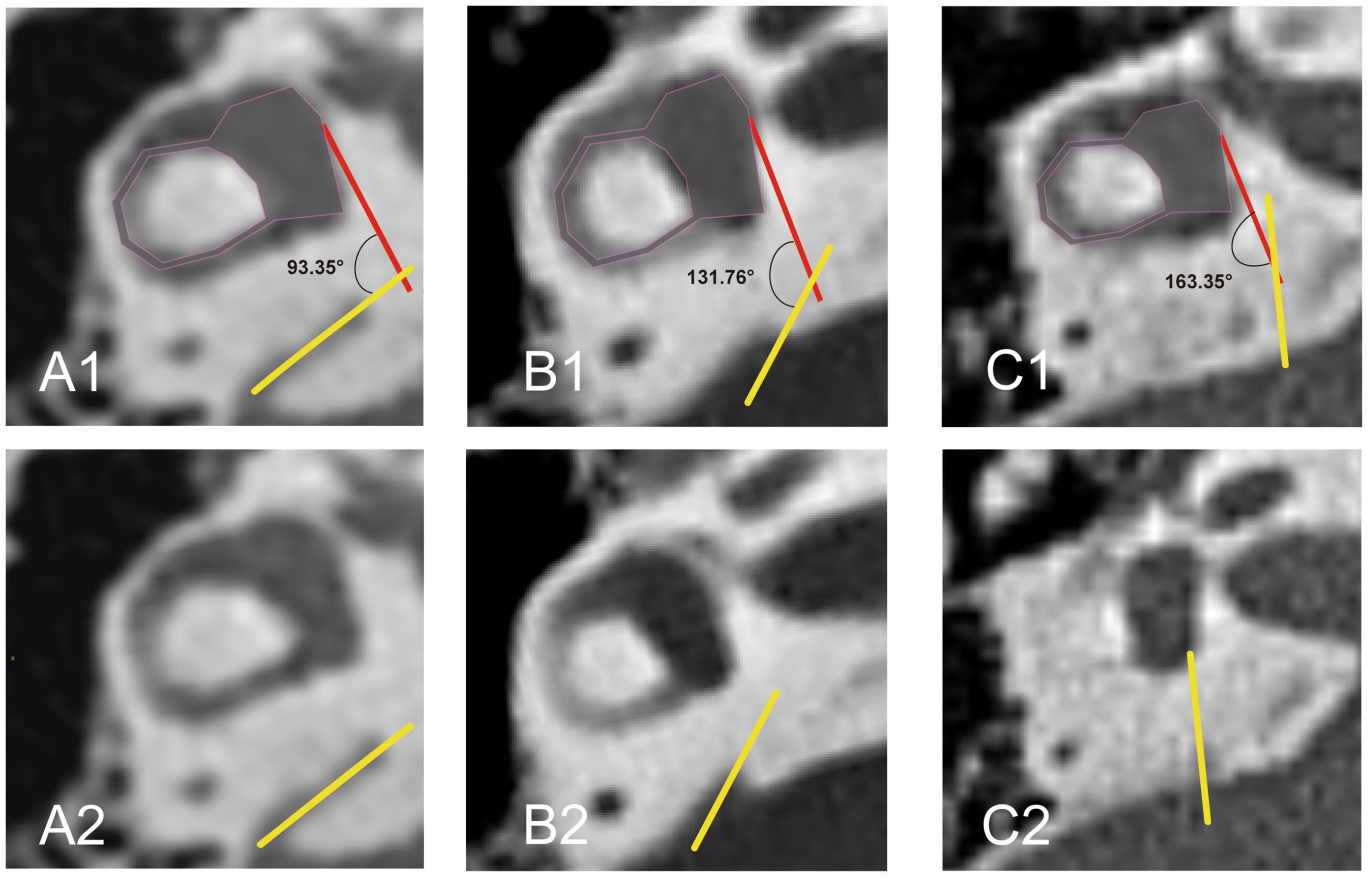
The measurement of the angular trajectory of the vestibular aqueduct (ATVA). The ATVA measurement of a 37-year-old male ipsilateral delayed endolymphatic hydrops patient **(A1,A2)**, a 36-year-old female control participant **(B1,B2)**, and a 52-year-old male Ménière’s disease patient **(C1,C2)** using two specific CT images, respectively. The angle between the red line and yellow line is the ATVA.

The measurement of ATVA was conducted following the protocol outlined by Bächinger et al. (9) via the link (http://links.lww.com/MAO/A755). A custom software tool, available for download at (https://github.com/DanielZuerrer/CoolAngleCalcJS), was employed for this purpose. As depicted in Figure 1, the initial image loaded into the software displayed the horizontal semicircular canal (SCC) and the vestibule. The software interface allowed for the adjustment of a magenta-shaded area to conform to the vestibule and horizontal SCC boundaries, particularly those of the vestibule, after which the first red line was established. The subsequent image imported into the software depicted the distal part of the VA, with a yellow line being aligned parallel to this section. The ATVA was calculated as the angle between the first red line and the second yellow line. Subsequently, the ears were categorized based on their ATVA measurements into three groups: those with ATVA ≤ 120°, those with 120° < ATVA < 140°, and those with ATVA ≥ 140°.

### 2.3 Videonystagmography

Vestibular tests were performed by using an infrared videonystagmography system (Visual Eyes VNG, Micromedical Technologies, Chatham, IL, USA) in accordance with a standard test protocol, including spontaneous nystagmus test, gaze test, saccades, smooth pursuit, and optokinetic test.

All DEH and MD patients underwent caloric test. Patients lay supine with head raised 30°, and their ears were alternately filled with warm and cold air (50 °C and 24 °C, respectively) for 40 s. The value of canal paresis (CP) was calculated based on the traditional Jongkes formula. CP value ≥ 25% is considered as caloric abnormality. The maximum slow phase velocity (SPVmax) of the caloric nystagmus was also measured. If the summed SPVmax was < 20°/s under four stimulation conditions, it is believed to indicate bilateral vestibular hypofunction. Ice water irrigation (4 °C, 1.0 mL) would then be used to confirm the caloric unresponsiveness.

### 2.4 Pure-tone audiometry

Pure-tone audiometry was conducted in a soundproof room, at 125, 250, 500, 1,000, 2,000, 4,000, and 8,000 Hz. PTA was calculated to be the simple arithmetic mean for frequencies of 500, 1,000, and 2,000 Hz.

### 2.5 Statistical analysis

The data were analyzed by SPSS 25.0 statistical software. Continuous variables were expressed as mean ± standard deviation or median (quartile). Categorical variables and grade variables were expressed as frequency (percentage). A Shapiro–Wilk test was used to test the normality of data. A t-test was used to compare continuous variables of two independent samples with normal distribution. A Mann–Whitney U test was used to compare grade variables or continuous variables of skewness distribution between two independent samples. Categorical variables were compared using Chi square test or Fisher’s exact test for two independent samples. *Post hoc* analysis was performed if necessary. The inter-rater reliability of the radiological results was determined using intra group correlation coefficient (ICC). The interpretation of agreement was as follows: ICC ≤ 0.20, poor; 0.2 < ICC ≤ 0.40, fair; 0.4 < ICC ≤ 0.60, moderate; 0.6 < ICC ≤ 0.80, good; 0.8 < ICC ≤ 1.0, excellent. Good to excellent inter-observer agreement was found in our radiological evaluation. Statistical significance was set as *p* < 0.05.

## 3 Results

In the present study, 27 ipsilateral DEH patients, 114 unilateral MD patients and 45 control participants were included. The age of ipsilateral DEH patients was 35.48 ± 15.78 year. The male/female ratio of ipsilateral DEH patients was 15/12. In the MD group, the age was 51(38, 57) year and the male/female ratio was 46/68. Stage I, stage II, stage III and stage IV were identified in 11/114 (9.6%), 22/114 (19.3%), 66/114 (57.9%), and 15/114 (13.2%) of MD patients. The PTA of DEH patients in the affected ear was 110.5 (103–120) dB, which was significantly higher than that of unilateral MD patients in the affected ear (54.0 ± 18.5 dB) (*Z* = −7.717, *p* < 0.001). Among DEH patients, 15 had profound hearing loss in childhood, and the duration between hearing loss and vertigo onset for the other 12 ranged from 1 to 19 years. The duration of hearing loss for MD patients was 1.55 (0.5–3) years

### 3.1 Prevalence of ATVA categories in patients and control

**Figure 2:**
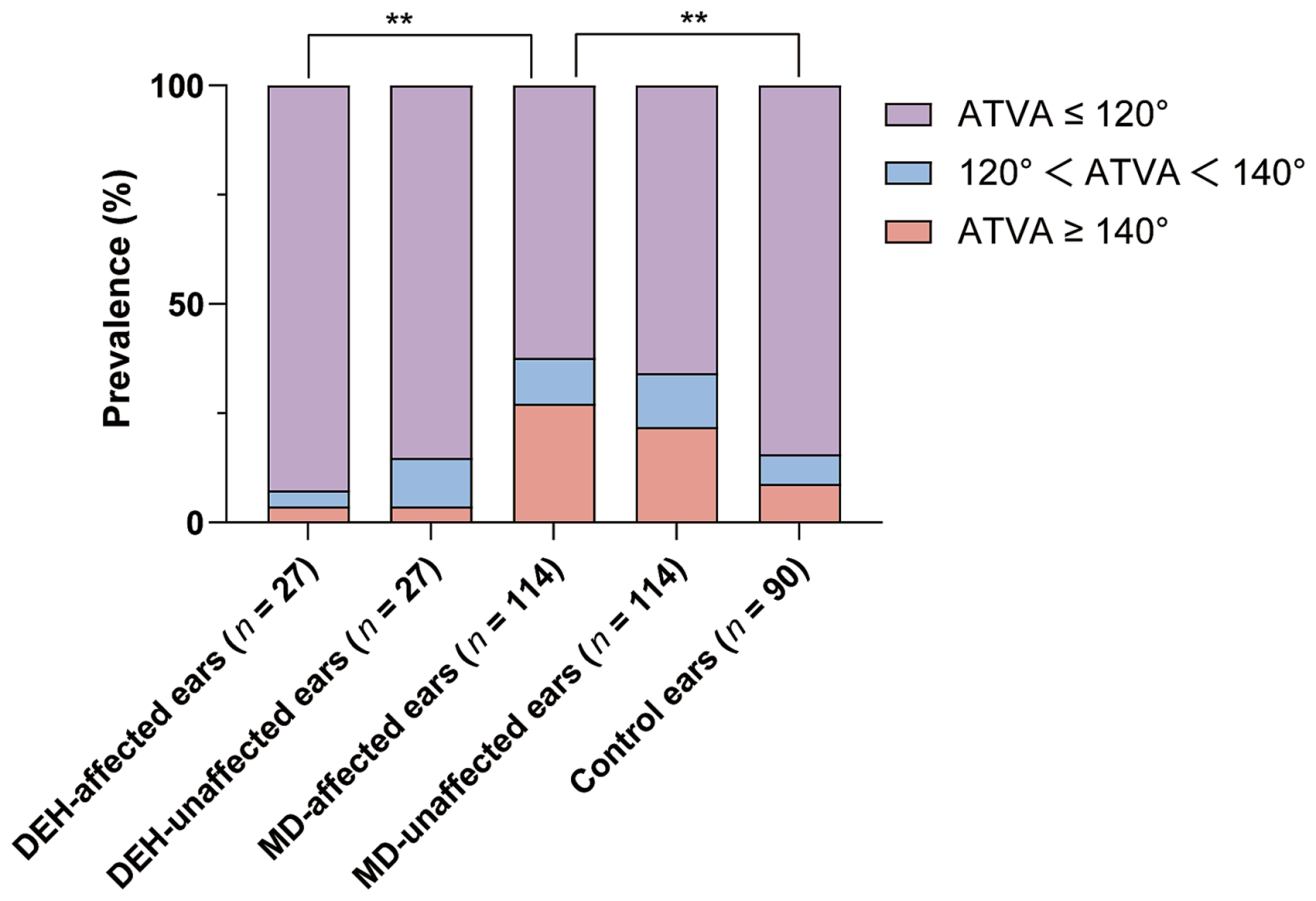
The distribution of the angular trajectory of the vestibular aqueduct (ATVA) categories in ipsilateral delayed endolymphatic hydrops (DEH) patients, unilateral Ménière’s disease (MD) patients and control participants. *n* = number of ears. **: *p* < 0.01.

As shown in Figure 2, the prevalence of ATVA ≥ 140°, 120° < ATVA < 140° and ATVA ≤ 120° was 1/27 (3.7%), 1/27 (3.7%) and 25/27 (92.6%) in affected ears of ipsilateral DEH patients, and 1/27 (3.7%), 3/27 (11.1%) and 23/27 (85.2%) in non-affected ears of ipsilateral DEH patients. In MD group, the prevalence of ATVA ≥ 140°, 120° < ATVA < 140° and ATVA ≤ 120° was 31/114 (27.2%), 12/114 (10.5%) and 71/114 (62.3%) in affected ears and 25/114 (21.9%), 14/114 (12.3%) and 75/114 (65.8%) in non-affected ears. ATVA ≤ 120°, 120° < ATVA < 140° and ATVA ≥ 140° were identified in 76/90 (84.4%), 6/90 (6.7%) and 8/90 (8.9%) of the control ears. The prevalence of ATVA category were statistically different among the above groups (χ^2^ = 25.836, *p* < 0.001). Bonferroni *post-hoc* comparisons were used for further analysis. (1) In both DEH and MD group, there was no significant interaural difference in the prevalence of ATVA categories (DEH group: χ^2^ = 1.083, *p* = 0.568; MD group: χ^2^ = 0.906, *p* = 0.636). (2) Significant difference was found between MD-affected ears and DEH-affected ears (χ^2^ = 9.355, *p* = 0.003). Compared to MD-affected ears, ATVA ≤ 120° was more prevalent (χ^2^ = 9.231, *p* = 0.002), whereas ATVA ≥ 140° was less frequent in DEH-affected ears (χ^2^ = 6.865, *p* = 0.009). (3) The prevalence of ATVA categories in DEH-affected ears was not significantly different from that of control ears (χ^2^ = 1.190, *p* = 0.510). (4) Significant difference was found between MD-affected ears and control ears (χ^2^ = 13.092, *p* = 0.001). Compared to control ears, ATVA ≥ 140° was more prevalent (χ^2^ = 10.897, *p* = 0.001), whereas ATVA ≤ 120° was less frequent in MD-affected ears (χ^2^ = 12.271, *p* < 0.001).

### 3.2 The relationship between clinical features and ATVA

**Figure 3:**
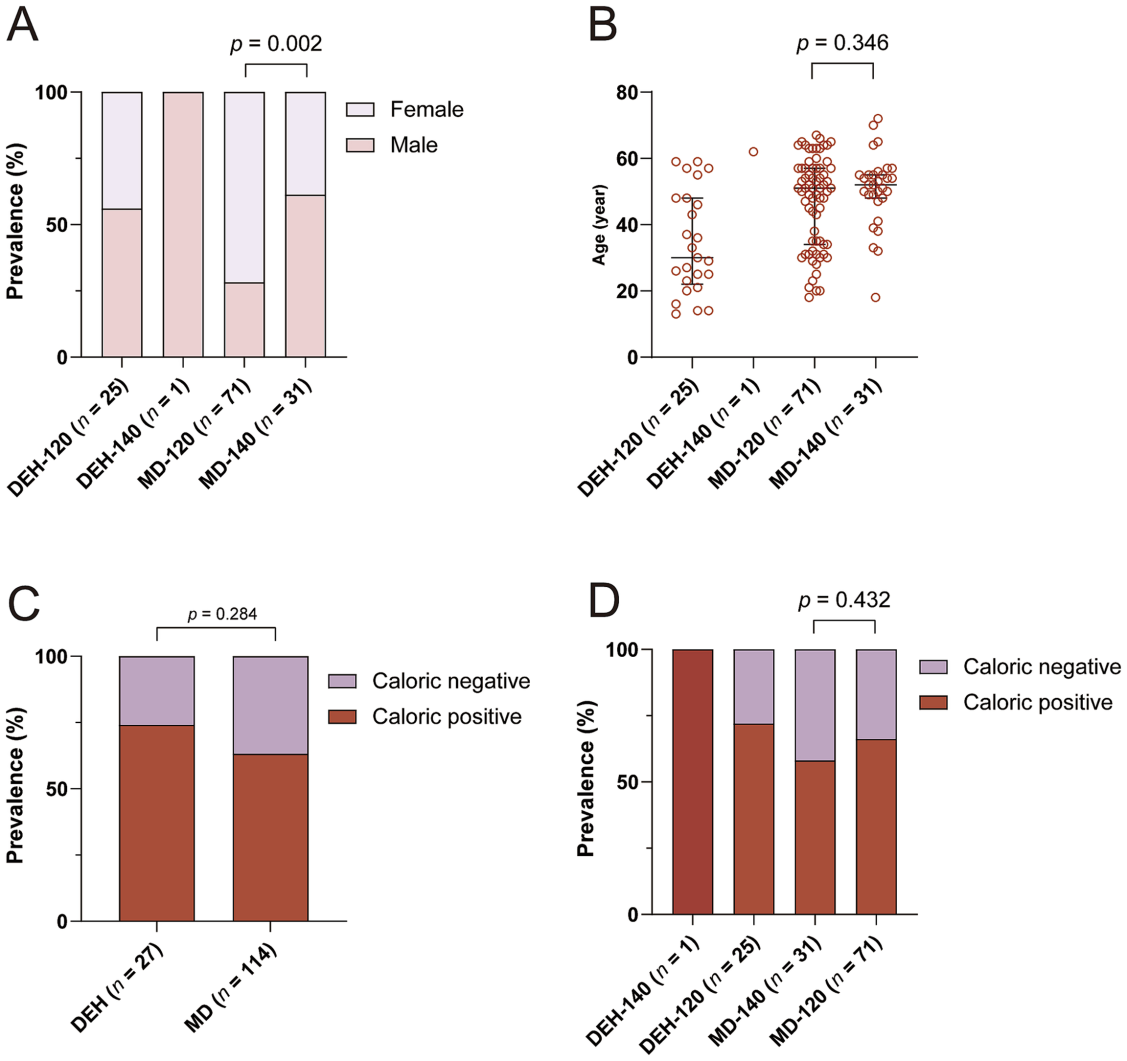
Comparison of clinical profiles between patients with ipsilateral delayed endolymphatic hydrops (DEH) and unilateral Ménière’s disease (MD). Comparison between DEH patients and MD patients of different angular trajectory of the vestibular aqueduct (ATVA) categories in terms of **(A)** Gender **(B)** Age. **(C)** Comparison between patients with DEH and MD in terms of caloric result. **(D)** Comparison between DEH and MD patients of different ATVA categories in terms of caloric result. *n* = number of ears.

Ipsilateral DEH patients with ATVA ≥ 140° and ATVA ≤ 120° on the affected ears were defined as DEH-140 and DEH-120, respectively. Male/female ratio in DEH-140 and DEH-120 were 1/0 and 14/11, respectively. The age of DEH-140 was 62 year, and the mean age of DEH-120 was 34.46 ± 15.16 year. Since only one DEH-140 case was enrolled in our study, intergroup comparison of proportions leads to unreliable statistical inference (Figure 3).

Unilateral MD patients with ATVA ≥ 140° and ATVA ≤ 120° on the affected ears were defined as MD-140 and MD-120, respectively. Male/female ratio in MD-140 and MD-120 were 19/12 and 20/51. Compared to MD-140 subgroup, female preponderance is significantly higher in MD-120 subgroup (χ^2^ = 10.024, *p* = 0.002). The age of the MD-140 and MD-120 were 52 (48, 55) and 51 (34, 57) year, respectively, which were not significantly different (*Z* = −0.943, *p* = 0.346) (Figure 3).

In the present study, all DEH patients and MD patients underwent caloric test and pure-tone audiometry test. As shown in Figure 3C, caloric abnormality was detected in 20/27 (74.1%) of ipsilateral DEH patients and 72/114 (63.2%) of unilateral MD patients, which was not significantly different (χ^2^ = 1.147, *p* = 0.284).

Caloric abnormality was found in 1/1(100%) DEH-140 and 18/25(72.0%) DEH-120 cases. The incidence of caloric abnormality was comparable between MD-140 (18/31, 58.1%) and MD-120 (47/71, 66.2%) subgroups (χ^2^ = 0.617, *p* = 0.432) (Figure 3).

## 4 Discussion

### 4.1 The prevalence of different ATVA categories in DEH and MD patients

In the current study, the prevalence of ATVA ≤ 120° in ipsilateral DEH-affected ears (92.6%) was higher than in MD-affected ears (62.3%), whereas the prevalence of ATVA ≥ 140° in ipsilateral DEH-affected ears (3.7%) was lower than in MD-affected ears (27.2%). This study, to the best of our knowledge, for the first time, found that the incidence of ATVA ≥ 140° in affected ears of ipsilateral DEH patients was significantly lower than that in unilateral MD patients. Our previous study also reported shorter PPD and lower VA visibility in MD patients than in ipsilateral DEH patients (20). Taken together, our present and previous results revealed that anatomical variations of the endolymphatic drainage system played differential roles in the pathogenesis of unilateral MD and ipsilateral DEH.

ATVA is a radiological parameter which indicates different pathological types of ES. Eckhard *et al*., in a histopathological study of temporal bone specimens from patients with MD and normal controls, found that MD is consistently correlated with either hypoplastic ES or degenerative ES (8). Histological and radiological assessment of the endolymphatic sac and vestibular aqueduct in MD patients by Bächinger et al. (9) discovered that hypoplastic ES was always associated with ATVA ≥ 140°, while degenerative ES was always associated with ATVA ≤ 120°. Further study found that the epidemiological features associated with MD-hp (predominantly men, familial clustering of MD, and tendency toward earlier disease onset), and the clinical features associated with MD-dg (a higher frequency of vertigo attacks and a more severe caloric response), support the hypothesis that the etiology of MD patients with hypoplastic ES (MD-hp) may be due to congenital factors, while the atrophic process in those with degenerative ES (MD-dg) may be caused by postnatal factors (8). Most recently, loss of function variant of PHEX gene, which is responsible for bone mineralization and phosphate homeostasis, has been identified as the causative gene for ES hypoplasia, MD and related audio-vestibular phenotypes, thus highlighting the congenital or genetic contribution associated with otic capsule mineralization in the development of MD in hypoplastic ES subgroup (26).

Our findings suggested that in ipsilateral DEH patients, degenerative ES may be the dominant histopathological type and postnatal factors may play a significant role. However, to date, there have been few pathologic studies in patients with DEH. Schuknecht et al. (27) have reported the pathology of two patients with contralateral DEH. Endolymphatic duct (ED) exhibited fibrotic obliteration with epithelial strand in case 1 and patent lumen in case 2. The ES were unremarkable without under- or mal-developed appearance (27). Future studies addressing the histopathological alterations of the temporal bone in DEH patients are needed to confirm our radiological findings.

Previous studies have suggested the role of various acquired factors in the development of DEH. Schuknecht postulated two preconditions for the development of DEH: (1) an initial labyrinthine insult of sufficient magnitude to cause total deafness but preservation of vestibular function, and (2) delayed atrophy or fibrous obliteration of the endolymphatic resorptive system (28). A recent imaging study using gadolinium-enhanced MRI (Gd-MRI) of the inner ear showed that the patients with profound deafness do not exhibit ELH *in vivo*, while the patients with DEH can exhibit ELH in vivo, implicating a “switch” factor that facilitates progression from profound deafness to DEH (29). Noise exposure, head trauma, and viral infections, *etc*. have been suggested as potential acquired causes of DEH (15, 30, 31). Autoimmune-related vestibular injury has been proposed to underlie DEH. In contralateral DEH, pathologic changes in the deaf ears were consistent with viral labyrinthitis and in the hearing ears the pathological changes were similar to those known to occur in MD, a mechanism resembling “sympathetic ophthalmia” (27). Suzuki et al. (32) found that 75% (6/8) of ipsilateral DEH patients showed at least one reactive band in the sera, which indicated the involvement of autoimmunity in DEH. Chen et al. (18) recently analyzed the results of high-throughput RNA- Seq and found differences in genetic profile between DEH patients and normal controls, such as MPZ, CLDN19, and PMP2, suggesting cell adhesion, neuropathy and autoimmunity are related to DEH. Factors other than anatomic variations, such as the immune-related mechanisms, need further investigations in DEH patients.

### 4.2 The prevalence of ATVA categories in DEH patients and control participants

In the present study, the prevalence of ATVA categories in DEH-affected ears was similar to that in control ears. Consistently, our previous study found no significant difference in PPD, an indirect indicator of ES size (19), between DEH patients and control participants (20). Bächinger et al. (9)found the prevalence of ATVA ≤ 120° was 62/62 (100%) in normal participants. In the study of Juliano et al. (33), the prevalence of ATVA ≤ 120° in control group was 59/61 (96.7%). In the research of Jung et al. (23), the prevalence of ATVA ≥ 140° in control group (patients without MD, or anatomic dehiscence of the superior canal) was 13/290 (4.4%). Robles-Bolivar et al. (34) found the prevalence of ATVA ≥ 140° in individuals without MD was 6/956 (0.6%). Compared to previous studies, we reported a relatively lower prevalence of ATVA ≤ 120° (76/90, 84.4%) in control group, which may be due to the limitations of imaging method. We speculate that the majority of patients with ipsilateral DEH have a normal or near-normal development of endolymphatic drainage system during infancy and early childhood.

### 4.3 The prevalence of ATVA categories in affected and non-affected ears of DEH patients

In this study, no interaural difference was found in the prevalence of ATVA categories in DEH patients. In our previous study, the prevalence of ATVA categories between affected and non-affected ears of MD patients was not significantly different (35). Lei et al. (20) also found no significant difference in PPD between affected ears (2.615 ± 1.659 mm) and non-affected ears (2.578 ± 1.345 mm) of DEH patients. Collectively, these studies indicate that neither ATVA nor PPD could serve as a radiological marker to determine the affected side, and besides anatomical variations, other factors may cause vestibular dysfunction in affected ears.

### 4.4 The relationship between clinical factors and ATVA in DEH patients

In the present study, ATVA ≥ 140° was found in only 1/27(3.7%) of DEH-affected ears, which may cause statistical bias when analyzing age, gender and caloric abnormality difference between DEH-140 and DEH-120. Bächinger et al. (11) found no significant difference in age between ATVA categories in MD patients, but a female predominance was found in MD-120, and the caloric responses in MD-140 and MD-120 were 30.2 ± 30.4% and 13.5 ± 15.2%, respectively, without significant difference. A larger sample size is needed to elucidate the relationship between ATVA and clinical features in DEH patients.

We found no significant difference in caloric abnormality between DEH patients and MD patients. In the study of Pollak *et al*., the result of caloric test was positive in 23/30 (76.0%) of DEH patients and 27/33 (82.0%) of idiopathic ELH patients, without significant difference (36). Previous researches revealed that the positive rate of caloric test was 76.5%—90.1% in ipsilateral DEH patients (37–39). Our result was in agreement with the above studies.

### 4.5 Strengths and limitations

To the best of our knowledge, this is the first study comparing the radiological differences in terms of ATVA measurements between patients with ipsilateral DEH and unilateral MD, both of which are classic hydropic ear diseases. Our study found that the prevalence of ATVA ≥ 140° and ATVA ≤ 120° in patients with DEH were different from those with MD, suggesting that the ES pathology in DEH is more consistent with the degenerative type rather than the hypoplastic type. These findings could deepen the understanding of the pathophysiological mechanisms of DEH and hydropic ear disease in a broader sense, and may help guide the formulation of treatment strategies.

This study had some limitations. Firstly, since DEH is a rare clinical entity, the inclusion of only one case in the DEH-140 subgroup limited further comparison between DEH subgroups and between DEH and MD groups. A larger sample size is required for further analyzing the relationship between ATVA and clinical features in DEH patients. Secondly, caloric test only measures the semicircular canal-driven angular VOR in very-low frequency range. The function of angular VOR in high-frequency range and the otolithic vestibulo-colic and -ocular pathway needs further investigation. Lastly, we did not perform Gd-MRI to validate ELH in neither DEH patients nor MD patients. Therefore, future studies should include a larger sample size and integrated imaging techniques and comprehensive vestibular function assessments for in-depth investigation. Moreover, histopathological studies of patients with DEH are warranted for future study.

## 5 Conclusion

Compared to MD patients, degenerative ES may be the dominant pathological type in DEH patients due to the high prevalence of ATVA ≤ 120°. The ELH in DEH patients may be largely due to degenerative factors.

## Data Availability

The original contributions presented in the study are included in the article/supplementary material, further inquiries can be directed to the corresponding author/s.
